# The Effect of Test Distance on Visual Contrast Sensitivity Measured Using the Pelli-Robson Chart

**DOI:** 10.1167/tvst.10.2.32

**Published:** 2021-02-19

**Authors:** Stevie M. Njeru, Mawada Osman, Angela M. Brown

**Affiliations:** 1 The Ohio State University, College of Optometry, Columbus, OH, USA

**Keywords:** Pelli-Robson (PR), Ohio Contrast Cards (OCCs), contrast sensitivity (CS), low vision, elderly

## Abstract

**Purpose:**

The Pelli-Robson (PR) chart is widely used to measure clinical contrast sensitivity (CS). It is generally believed that PR testing distance is not critical. Here, we examine whether a closer test distance than the usual 1 meter might be better for patients with low vision.

**Methods:**

PR CS was measured on two groups: low-vision students (<20 years old) and elder patients (>65 years old). Student PR was measured at 1 meter and at a closer distance d = visual acuity in log_10_cy/deg (d = 1.5–logMAR). Elder PR was measured at 1 and 3 meters. Grating CS was also measured using the Ohio Contrast Cards (OCCs).

**Results:**

Average CS was 0.398 log_10_ units (over one line on the PR chart) higher at the closer distance than at 1 meter for the students, but there was no effect of 1 vs*.* 3 meters test distance for the elders. The equivalent spatial frequencies of the PR letters at 1 meter were near the acuity limits of students with low vision, but were near the peak of the elders’ CS functions. Especially for students with low vision, PR CS was below OCC CS, even when PR was tested at a closer distance.

**Conclusions:**

PR CS should be measured at a distance in meters that is equal to the patient's letter acuity in cy/deg, or 1.5–logMAR.

**Translational Relevance:**

Contrast sensitivity is highly associated with quality of life, and it is important to measure it accurately. Using a closer distance, or measuring grating CS, can reveal visual abilities missed when patients with low vision are tested using PR at 1 meter.

## Introduction

When an ophthalmologist or optometrist needs to measure a patient's contrast sensitivity, they usually use the Pelli-Robson (PR) contrast sensitivity chart.^1^ This chart is typically viewed from a distance of 1 meter, where the letters subtend 2.85 degrees of visual angle. It is generally believed that testing at this distance is good for a wide range of patients because the patient can use the fundamentals or the harmonics of the letters’ Fourier spectra, as needed, to recognize and identify the letters. The Mars contrast sensitivity chart^2^ is designed on similar principles, to be used at a distance of 40 to 50 cm. However, these prescribed test distances will not be appropriate for many patients with low vision. For example, the PR letters are 20/672 (1.53 logMAR) at 1 meter, so the chart obviously cannot be used at 1 meter to test a patient whose visual acuity is worse than that. Yet, it is precisely in a low-vision context that the PR chart is most likely to be useful clinically. Hopkins, Dougherty, and Brown^3^ proposed that a better test distance could be chosen based on the patient's letter acuity. In this study, we investigated the impact of testing patients with low vision at a closer distance. For comparison, we also investigated the impact of testing a group of elderly primary vision care patients without severe visual acuity deficits at 3 meters, the distance originally recommended by Pelli, Robson, and Wilkins.^1^ This group of participants was chosen to provide baseline data for a future project on contrast sensitivity testing of patients with dementia.

## Methods

### Participants

These tests were carried out on two groups of participants. At the Ohio State School for the Blind (OSSB), 30 schoolchildren with low vision (students) participated in this study. Students were identified by OSSB as being “partially sighted” and were recruited by postal letter. They were 7 to 20 years old (mean age = 15.1, SD = 3.7, mean grade level = 8.0, SD = 3.9). After hearing a full explanation of the study, students participated with the informed permission of their parents and with their own assent, or, for those over age 18 years, with their own informed consent. The assent/consent forms were read to the students, as none could read the printed forms visually. At the Primary Vision Care (PVC) service of the Ohio State University College of Optometry, we tested 46 ambulatory adult patients (“elders”) over 65 years old (mean = 72.0 years, SD = 6). They were recruited when their PVC appointments were scheduled, and they were tested immediately before their regular eye examinations. Those who arrived with caregivers participated with the permission of the caregiver and with their own assent after the study had been fully explained; others participated with their own informed consent. The visual disorders of the participants appear in Appendix A.

This study was approved by Ohio State University's Institutional Review Board for protection of human subjects, and it adhered to the principles of the Declaration of Helsinki.

### Procedures

This protocol was designed to compare PR contrast sensitivity tested at 1 meter (PR1) and PR contrast sensitivity measured at another distance: a closer distance (PRclose) for the OSSB students, 3 meters (PR3) for the elders. We also measured visual acuity using a letter chart, and we measured contrast sensitivity using the Ohio Contrast Cards (OCCs). Testing was monocular, using the participant's preferred or only sighted eye, except for two OSSB students who were tested binocularly because they refused the eye patch. All patients wore their habitual refractive correction.

Testing at OSSB began with a Bailey-Lovie (BL) chart measurement of visual acuity, followed by the PRclose measurement by the same examiner. The BL acuity was always measured first because the closer test distance for the PR chart depended on the patient's logMAR acuity, as outlined below. At PVC, the first examiner performed the PR3 measurement. At both sites, a second examiner performed the PR1 measurement, using a PR chart with different letters, and the OCCs. The examiners at OSSB were coauthors S.M.N. and A.M.B., and the examiners at PVC were coauthor M.O. and assistant F.O.O. The assignment of test groups to examiners was randomized across participants, and the second examiner was always unaware of the first examiner's results. At OSSB, the uniform illumination was 735 lux (BL and PRclose) or 745 lux (PR1 and OCC), and at PVC the uniform illumination was 590 lux (PR3) or 490 lux (PR1 and OCC). The space-averaged luminance levels at OSSB were: 234 cd/m^2^ (BL and PRclose), 237 cd/m^2^ (PR1) and 119 cd/m^2^ (OCC, because the reflectance of the gray card was 50%). At PVC, these were: 188 cd/m^2^ (PR3), 156 cd/m^2^ (PR1), and 78 cd/m^2^ (OCC). Rovamo et al.^4^ showed that contrast sensitivity, measured with 0.25 cy/deg and 1 cy/deg sine-wave gratings, is essentially constant with luminance above 10 cd/m^2^.

### Vision Tests

#### Letter Acuity

For the students with low vision who could read letters, letter visual acuity was measured using a printed BL LogMAR chart (National Vision Research Institute of Australia, prepared by the Multimedia Center, School of Optometry, Berkeley, CA). The test distance was typically 2 meters, but it could be adjusted if necessary, with the distance compensated for in the letter-by-letter scoring of visual acuity. For the elders, letter acuity was taken from their health records at the PVC service, where it was measured clinically using the ClearChart-4 digital acuity system (AMTEK Technologies, Inc., Depew, NY). The system was set up to present the letters at 220 cd/m^2^, and the viewing distance was 20 feet.

#### Pelli-Robson Contrast Sensitivity

The PR chart presents letters in two groups of three (two “triplets”) in each row, in descending order of contrast. The letter contrast was constant within triplets, and each triplet differed from its predecessor by −0.15 log_10_ units. Each row (and each letter within a column) differed from the one above it by 0.301 log_10_ units. The PR chart test was administered by asking the participant to read a vertical column of letters, one letter from each line, until they hesitated to identify a letter or until an error occurred. Then the participant read the letters by line, starting at the previous line, and continuing until two mistakes were made within a triplet of letters. Scoring was letter-by-letter, and log_10_ letter contrast was log_10_ ([W–D]/W), where W is the reflectance of the white surrounding field and D is the reflectance of the dark letters. The space-average luminance of the PR chart was about W. All test distances were measured using a tape measure, and once chosen, were maintained throughout testing.

When testing students with low vision using the PR chart^1^ (Precision Vision, Inc.), the closer test distance was chosen based on the formula from Hopkins et al.^3^ This formula was based on the equivalent spatial frequency of the PR letters compared to their modeled contrast sensitivity function (CSF) (see their figure 7). The formula was:

(1) $$\begin{array}{c} d=1.5-\mathrm{VAa}, \end{array}$$

or equivalently,

(2) $$\begin{array}{c} d∼\mathrm{VAb}. \end{array}$$

**d** is the prescribed test distance in meters, **VAa** is the letter visual acuity in logMAR units, and **VAb** is the letter visual acuity in log_10_ cy/deg, which is calculated as log_10_(30)–logMAR. Students with **VAa** > 1.25 logMAR (**VAb** worse than 0.23 log_10_ cy/deg) were allowed to approach the chart as closely as they liked for their closer distance. Notice that the units are not “correct” in these formulas, as **d** is in linear meters whereas VAa and VAb are log_10_ units related to cy/deg. Therefore, these formulas are provided as a rule of thumb for the convenience of the examiner, but they have no particular theoretical justification beyond the fact that they prescribe a test distance that places the PR letters at a spatial frequency that is about 1.5 log units lower than the letter acuity cutoff.

For the elder participants, the PR test distance was either 1 meter or 3 meters.

#### Ohio Contrast Cards

Contrast sensitivity in log_10_ units was also measured on all participants using the OCCs^3^ (Precision-Vision, Inc.) using the card protocol^5^^,^^6^ originally designed for the Teller Acuity Cards, at a test distance of 57 cm. The OCC test presents 3 cycles of a 20-cm square, 0.15 cy/cm horizontal square-wave grating, and each card's grating contrast differed from its predecessor by -0.15 log_10_ units. Grating contrast was (L–D)/G, where L is the reflectance of the light bars, D is the reflectance of the dark bars, and G is the space-average luminance of the cards, i.e. half the maximum possible value of L. This definition is preferred over the Michelson contrast ([L–D]/W), which is more usually used for gratings,^7^ to make the OCC grating contrast more directly comparable to the contrast of the PR chart letters.

### Cognitive Status of Elder Participants

After all the vision tests were completed on an elder participant, the second examiner evaluated their cognitive status using the Six-Item Cognitive Impairment Test (6-CIT). The 6-CIT^8^ was chosen because it is brief and has been validated for use on elderly participants. For high test sensitivity, an elder was considered cognitively competent for this research if the 6-CIT score was 7 or lower.^9^

## Results

### Completed Tests

Twenty-one of the 30 students with low vision provided complete data sets. Three students with septo-optic dysplasia were unable to perform any of the tests. Except for these three, every student completed the OCCs. An additional six students provided incomplete letter chart data. Each analysis was performed using all the available data.

Thirty-nine of the 46 elder participants provided complete data sets. One of the elders was unable to perform the PR chart at 3 meters because her visual acuity was 1.0 logMAR, whereas the PR3 letters were at 1.05 logMAR. Six elders failed the 6-CIT cognitive test with a score ≥8. The data from those seven elders were eliminated from analysis.

### The Effect of Test Distance on Pelli-Robson Contrast Sensitivity

#### Test Distances

**Figure 1.**
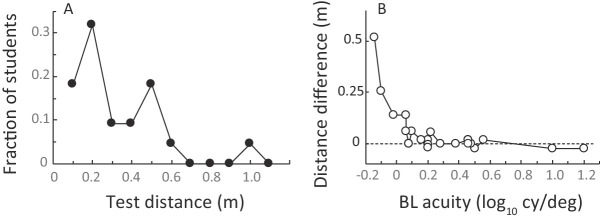
“Close” distances for low-vision students. (**A**) Distribution of test distances; (**B**) the difference between the prescribed test distance **d** (Equations 1 and 2) and the actual test distance.

In the PRclose condition, students were tested at a median distance of 24 cm (range = 0.1 meter to 1.0 meter; Fig. 1A). For 17 of the 21 students with low vision who were successfully tested at both 1 meter and close distances, the closer test distance was within 6 cm of **d** (Equations 1 and 2). Only 4 students, all with **VAa** > 1.41 logMAR (**VAb** worse than 1.17 cy/deg), chose test distances that were more than 6 cm farther from the chart than their personal value of **d** (Fig. 1B).

#### Contrast Sensitivity

**Figure 2.**
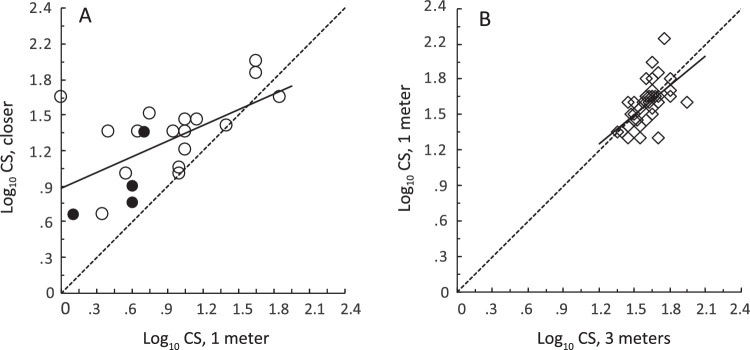
The effect of test distance on Pelli-Robson contrast sensitivity. *Dotted lines:* equality. Continuous regression lines were fitted to the data. (**A**) Contrast sensitivity of students with low vision at the closer distance, as a function of their contrast sensitivity at 1 meter. *Solid circles:* students who were tested at more than **d** meters from the chart. (**B**) Elders’ contrast sensitivity at the closer distance (1 meter), as a function of their contrast sensitivity at the farther distance (3 meters).

**Figure 3.**
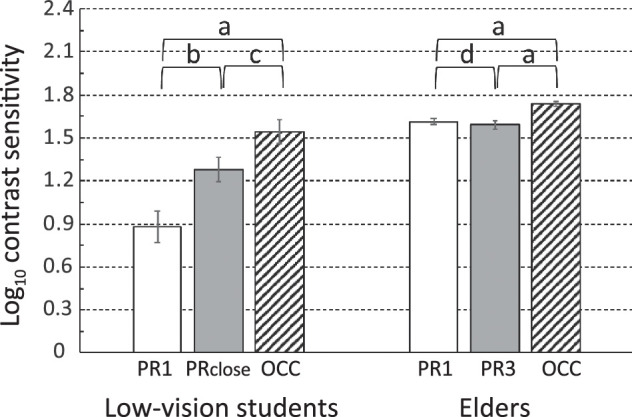
Comparison between contrast sensitivity measured using the PR at 1 meter (*white bars*) and at another distance (*gray bars*) and using the OCC (hatched bars), ±1 standard error of the mean. “a”: *P* < 0.0003; “b”: *P* = 0.0006; “c”: *P* = 0.007, “d”: *P* > 0.5, n.s. Horizontal lines mark 0.3 log_10_ units, the contrast difference between two adjacent rows on the PR chart.

The average contrast sensitivity (CS) of students with low vision was 0.398 log_10_ units higher when tested at the closer distance than at 1 meter (Figs. 2A, 3). This is an improvement by a factor of 2.5, or over one line of 6 letters on the PR chart. A paired *t*-test showed that this difference was statistically significant (t_20_ = 4.57, *P* = 0.0002), and linear regression analysis showed that the two PR measures were significantly correlated with one another (*r* = 0.602, *P* = 0.004). The results from the four students who did not sit as close to the chart as was prescribed by Equations 1 and 2 (filled circles in Fig. 2A) were unremarkable and were included in this analysis. Hopkins et al. estimated that the students in their study would have improved their contrast sensitivity by 0.36 log_10_ units, had they been able to view the PR chart from the optimum distance. The present improvement of 0.398 log_10_ units agreed with Hopkins’ estimate (t_20_ = 0.432, *P* = 0.670, NS).

For the elders, there was no reliable difference between CS measured at the two distances (t_37_ = 0.799, NS; see Figs. 2B, 3). However, there was a statistically significant correlation between the PR contrast sensitivity values at the two distances (*r* = 0.558, *P* < 0.0001).

#### Letter Versus Grating Tests of Contrast Sensitivity

Figure 3 compares the grating card and letter chart contrast sensitivities for students with low vision and elders. OCC grating cards revealed higher contrast sensitivity than the PR letter chart, at both test distances, for both groups of participants. Paired *t*-tests showed t > 3.4 and *P* < 0.01 for those comparisons (labeled “a,” and “c” in Fig. 3), after Bonferroni correction. Statistical power analysis, assuming standard deviations from the prior literature^10^ and a minimum difference of 0.15 log_10_ contrast, showed that there were not enough patients in any diagnostic group (Appendix A) to compare these results across diagnoses within either the elder or the student participant groups. Using the standard deviations of the present data on our elderly participants, post hoc, there were sufficient patients with aphakia, patients with cataract, and patients with no cataract history within the elderly group to compare them. A multivariate analysis of variance showed that neither PR3 nor PR1 nor OCC CS values varied across those three diagnoses (Wilks’ Lambda = 0.286).

To compare the levels of contrast sensitivity intuitively, the graph in Figure 3 is marked off (dashed lines) in 0.3 log_10_ unit steps, the difference between subsequent full lines of letters on the PR chart. For the students, contrast sensitivity measured by the PR at 1 meter was 0.662 log_10_ units below that measured by the OCC, a difference of over 2 lines on the PR chart. Even at the closer distance, the PR value was 0.265 log_10_ units, or over 5 letters (almost a full line on the PR chart), below the OCC value. For the elders, these differences were smaller (less than a triplet on the chart).

### Visual Acuity

**Figure 4.**
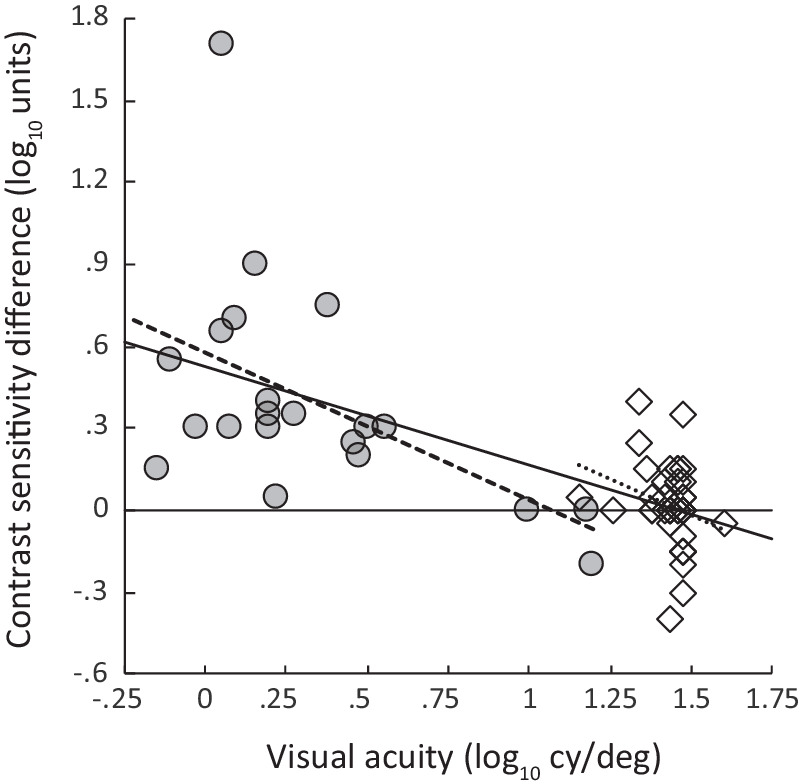
Difference between PR contrast sensitivity at two distances as a function of letter visual acuity. Students with low vision: PRclose – PR1 (*gray circles*, *dashed line*; *r* = 0.493, *P* = 0.023). Elders: PR1 – PR3 (*white diamonds*, *dotted line*; *r* = 0.258, *P* = 0.117, n.s.). *Bold solid line*: regression line drawn through both data sets (*r* = −0.659, *P* < 0.00001).

Figure 4 shows the difference between patients’ CS at 1 meter and their CS at the other test distance (compare the white and gray bars in Fig. 3) as a function of the patient's visual acuity as measured using letter charts, for the two groups of observers, along with the associated regression lines and their statistical significance. Clearly, the advantage of testing at a close distance was related to the patient's visual acuity, as predicted by Hopkins et al. Considering all the data together, the worse the patient's visual acuity measured using letters, the more important it is to test at a distance closer than 1 meter.

## Discussion

The goal of this project was to determine whether performance on the PR chart could be improved by simply adjusting the physical distance between the patient and the chart, as suggested by Hopkins et al.^3^ (Equations 1 and 2). We tested that prediction directly for a group of students with low vision, who were similar to the students studied by Hopkins et al. We found that performance on the PR test did indeed improve when the students with low vision viewed the chart from a closer vantage point. By comparison, we found little difference between the CS performance at 1 meter and 3 meters among our elder participants.

### PR Contrast Sensitivity and the Contrast Sensitivity Function 

To examine the basis for these seemingly discrepant results between the students with low vision and the elders, we needed a way of comparing the letter data to the spatial CS of the visual system, specified relative to a spatial frequency abscissa. We took the center frequency of the spatial-frequency-tuned channel used to detect a letter to be its “equivalent” spatial frequency, calculated using the formula from Majaj, Pelli, Kurshan, and Palomares^11^ (see also reference 12). Then, we adjusted the CSF template from Chung and Legge^13^ to pass through the average of the transformed PR and letter acuity data for each group of participants. We chose the Chung and Legge CSF template over other alternatives because those authors have validated its invariant shape when fitted to data from participants with normal and low vision with central field loss.

**Figure 5.**
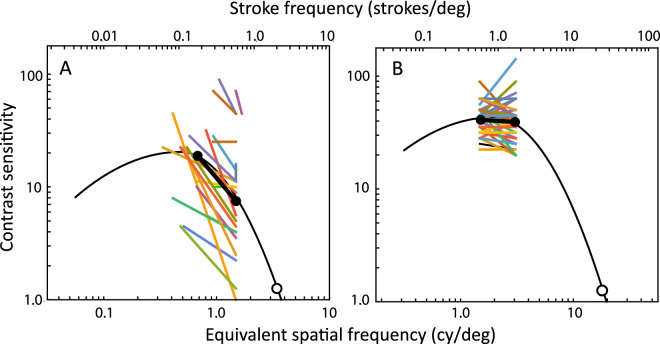
Individual participants’ PR contrast sensitivity values (*colored lines*) compared to the CSF of the visual system (*black curves*), fitted as a template to the average PR contrast sensitivity (*black circles and bars*) and average letter visual acuity (*white circles*). Lower axis, the spatial frequency of the channel used to identify the letter. Upper axis, the spatial frequencies of the strokes comprising the letters, based on their logMAR values. See Majaj et al.^11^ for details of the conversion between the upper and lower abscissas. (**A**) Students with low vision; (**B**) elders.

The result of this analysis (Fig. 5) is clear: the PR letters, when viewed by students with low vision at a distance of 1 meter (where their spatial frequency is equivalent to 1.48 cy/deg), are on the falling high-frequency end of the CSF. The closer test distances for the PRclose condition moved the equivalent spatial frequencies of the letters closer to the maximum of the students’ CSF, and those distances produced a clear improvement in their PR CS performance. The story was quite different for the elders. Their data fell near the peak of the CSF, so a factor-of-three change in the test distance did not have any great effect on the measured PR chart performance. Notice that Equations 1 and 2 predict a test distance of 1.5 meters for the normal patient (logMAR = 0). This is within the range we tested, it is near the distance suggested by the PR manual, and it is right at the peak of the normal CSF.

Visual acuity and CS can be correlated across patients for two reasons. One is because many disorders affect both aspects of visual function, resulting in the CSF being displaced simultaneously in two dimensions, relative to the spatial frequency and the contrast sensitivity axes (e.g. reference 13). The other is because of statistical regularities that result when the displacement component along one dimension affects the measured level of the other dimension. The biggest effect is that the leftward lateral component of the displacement of the CSF will necessarily cause a large loss in CS on the right-hand edge of the function, where the curve is steepest. However, the leftward component of CSF translation in two dimensions will not produce very much loss of sensitivity near the maximum of the CSF, where the curve is nearly flat.

Here, the data point measured at 1 meter on the students was well down on the falling end of the CSF, whereas the data point measured at the closer distance was nearer the peak of the CSF. Therefore, the lateral component of the two-dimensional CSF displacement due to their disorders had a major effect on the level of PR CS measured at 1 meter, resulting in a strong correlation between visual acuity and PR1 contrast sensitivity (see Fig. 4). The lateral component of the CSF displacement had less effect on the level of PR CS measured at the closer distance, and there was a weaker correlation between visual acuity and PRclose CS. This suggests that an advantage to testing patients with low vision at the PRclose distance is that PR CS will be relatively unaffected by the patient's visual acuity.

The story is quite different for the elders. At 1 meter and 3 meters, their PR data points are both near the maximum of the CSF. Therefore, the vertical and horizontal components of the displacement of their CSFs as a result of their visual disorders will have separate effects on the measured values of PR CS and letter acuity. However, there is no interaction between these effects: the lateral component of the displacement does not directly affect the measured CS.

### OCC Versus PRclose

Even at the closer test distances, CS was better when measured using the OCC compared to the PR chart (compare hatched bars to gray bars in Fig. 3). In this report, the PR letter contrast was expressed (conventionally) as a Weber fraction (i.e. the luminance modulation divided by the overall luminance of the stimulus). The OCC grating contrast was also expressed (unconventionally) as a Weber fraction, in order to make the contrast levels of the two stimuli as comparable as possible. If the contrast of the grating had been expressed in the usual Michelson units, the difference would have seemed even greater (by a factor of 2). Therefore, the unusual choice of Weber contrast units for the OCC was not responsible for the modestly higher CS measured using the OCC compared to PRclose.

This difference is also probably not wholly due to the larger size of the grating (20 degrees tall) compared the letters at the closer distance (2.85 degrees tall for the elders, and 11.9 degrees tall on average for the students). Stimulus area has a powerful effect on contrast sensitivity,^14^ even among visually normal observers. However, the difference in area between the grating and the letters was smaller (an area factor of 2.8) for the students, who showed a somewhat larger CS difference, and much greater (an area factor of 49) for the elders, who showed a somewhat smaller CS difference. This difference between OCC and PRclose is probably not directly related to the cognitive demands of identifying letters either, because it is present for both the students, who were highly variable in both age and cognitive ability, and also for the elders, all of whom were competent readers.

However, the relative sizes of the stimuli and the difference between the detection and identification tasks may be important when considered together, and these differences may be important clinically. The PR letter identification task requires the observer to see spatially localized features that are present or absent in different locations within the area of each letter stimulus. Thus, a small area of reduced sensitivity (either at the fovea or elsewhere in the visual field) could impair the PR letter identification performance of a patient with low vision, even if the rest of the visual field is intact. By comparison, the OCC grating covers a large area of the visual field, any part of which will be sufficient for detection. This might account for the higher CS measured using the OCC gratings, and it might also make OCC gratings relatively immune to small scotomas or other variations in sensitivity across the visual field. It could also improve the ability of the OCC to describe the overall visual capabilities of the patient with low vision. To interpret these results further, research will be needed to link OCC CS to vision disorders and to the patient's function in everyday life (see Hopkins et al.^3^ for a small study of quality of life).

### Other Distances, Other Tests

We do not know empirically whether even closer distances would increase or reduce the level of CS measured using the PR chart. It seems likely, based on the work of Majaj et al., that if even closer distances are used, patients would use other Fourier components of the letters to perform the task, resulting in no major loss of performance if the patient is “too close” (see also Zhang, Pelli, and Robson, 1989, cited in reference 7). However, even those patients with visual acuity worse than 1.25 logMAR chose test distances within 6 cm of the distance predicted from Equations 1 and 2, unless their visual acuity was 1.41 or worse, at which point the equations would give near-zero of negative test distances. This agreement between the predicted and chosen distances suggests that the equations agree with the lived reality of vision experienced by these patients. It also suggests that the patients themselves may know from their own experience how close they need to be to read the letters, so a strategy of letting the patient choose the distance might work well (while recording the chosen test distance, of course), and might relieve the clinician of the task of estimating the distance for each patient. Further work would be required to validate this ad libitum testing strategy.

It would be difficult to apply this strategy to the Mars chart,^2^ a smaller, handheld CS letter chart. The Mars chart letters, when viewed at 35.7 cm distance, subtend 2.8 degrees at the eye, the same angular subtense as the PR letters at 1 meter. To make the Mars chart letters as large (at the eye) as the PR letters were for the students with low vision at the close distances used here, the Mars chart would have to be viewed from a median distance of 8.6 cm. Twenty-five percent of our participants would need to place the Mars chart as close as 6.4 cm. This would pose obvious problems, such as the patient's head interfering with the light, the additional “plus” refractive correction required, and the absolute need for monocular viewing, because even a visually normal observer would have difficulty verging the eyes at that close distance.

## Conclusions

When using the PR chart, test distance is more important than is generally believed. Although still not as good as the performance revealed by the OCC, the performance of school-aged students with low vision (but not the performance of elders who seek primary vision care) can be improved by a factor of 2.5 by simply adjusting the test distance based on the student's letter acuity.

## Figures and Tables

**Figure A1.**
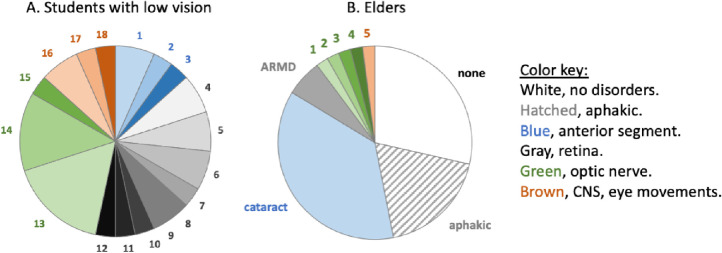
Participant disorders.

## Appendix A.

**Table A1.** Key to Figure A1-A

Students With Low Vision		
Disorder	Key	*N*
Congenital cataracts	1	2
Axenfeld-Rieger syndrome	2	1
Stickler syndrome	3	1
Blue cone monochromat	4	2
Rod-cone dystrophy	5	2
Retinopathy of prematurity	6	2
Albinism	7	1
Central areolar macular dystrophy	8	1
Congenital glaucoma, Aniridia	9	1
Leber's congenital amaurosis	10	1
Retinitis pigmentosa	11	1
Retinoblastoma	12	1
Optic atrophy	13	5
Septo-optic dysplasia	14	4
Optic nerve hypoplasia	15	1
Cortical blindness	16	2
Congenital nystagmus	17	1
Leukemia	18	1

**Table A2.** Key to Figure A1-B

Elders		
Disorder	Key	*N*
None	None	14
Cataract	Cataract	9
Aphakic	Aphakic	18
Age-related macular degeneration	ARMD	3
Benign neoplasm of choroid	1	1
Glaucoma	2	1
Lamellar macular hole	3	1
Macular pucker	4	1
Progressive supranuclear ophthalmoplegia	5	1
